# Public and Patient Involvement in Doctoral Research During the COVID-19 Pandemic: Reflections on the Process, Challenges, Impact and Experiences From the Perspectives of Adults With Cerebral Palsy and the Doctoral Researcher

**DOI:** 10.3389/fresc.2022.874012

**Published:** 2022-06-03

**Authors:** Manjula Manikandan, Kevin Foley, Jessica Gough, Sarah Harrington, Éabha Wall, Fiona Weldon, Jennifer M. Ryan, Claire Kerr, Aisling Walsh, Jennifer Fortune

**Affiliations:** ^1^Department of Public Health and Epidemiology, RCSI University of Medicine and Health Sciences, Dublin, Ireland; ^2^Public and Patient Involvement Contributor, Ireland; ^3^College of Health, Medicine and Life Sciences, Brunel University London, Uxbridge, United Kingdom; ^4^School of Nursing and Midwifery, Queen's University Belfast, Belfast, United Kingdom

**Keywords:** Public and Patient Involvement (PPI), COVID-19, adults, cerebral palsy, doctoral research, perspective

## Abstract

**Introduction:**

Cerebral palsy (CP) is a lifelong condition, where people may experience complications as they age. Including the views of people with CP through Public and Patient Involvement (PPI) ensures that research into the condition is relevant and meaningful in addressing their concerns. However, there is a lack of evidence on incorporating the voices of adults with CP in the doctoral research process. Therefore, this paper aims to provide an overview of how adults with CP were involved in a doctoral research process during the pandemic.

**Methods:**

This paper describes the PPI process and its impact at various stages of the doctoral research process and reflects on the experiences from the perspective of the doctoral researcher and adults with CP using the INVOLVE Values and Principles framework. Five adults with CP were consulted throughout the doctoral research programme. The data for this paper is a combination of reflection notes, email exchanges, meeting minutes and informal discussions with the PPI team on their experiences of being involved in the PPI process. The content of this paper is informed by GRIPP 2 checklist.

**Results:**

The doctoral researcher and adult reflections highlighted the value of collaboration and the positive impact on research at each stage of the doctoral research process. Although meetings were adapted due to the pandemic, the values of PPI were adhered to throughout the doctoral research.

**Conclusion:**

Involving adults with CP positively impacted the doctoral research process. It is recommended to consider individual access needs to ensure meetings and information are accessible for disabled adults. Our reflective findings and recommendations may help other researchers who plan to involve adults with CP in doctoral research.

## Introduction

Public Involvement in research is defined as “research being carried out ‘with' or ‘by' members of the public rather than ‘to,' ‘about' or ‘for' them.” (1). It is well documented that Public and Patient Involvement (PPI) ensures that research is relevant and meaningful in addressing issues that concern the population being studied (2, 3). The term ‘public and patient' refers to people who are experts by experience and can include people with lived experience of a condition, and with an interest and experience in using health and social care services (1). Involvement should be a collaborative journey with adequate funding, training, time and additional staff to coordinate, facilitate and build a trusted relationship between public contributors and researchers (4, 5). Involvement in doctoral research was reported to enhance doctoral researcher's confidence and credibility of research, improve study progression, recruitment, analysis, relevance or quality of research, implementation and dissemination of findings (6–10). However, PPI in the doctoral research process can be challenging for researchers who often have limited funding, resources, and time to conduct meetings or build relationships. There may also be limited training available for doctoral researchers (6–8). Another challenge that was identified in the literature is managing the expectations of multiple stakeholders by the doctoral researcher. Stakeholders may include the supervisory team, research funders, University, and PPI contributors (6–8). In addition, doctoral researchers are required to take ownership of their research process, but may not have adequate training to manage expectations or to take ownership of their research.

Recent articles have described the experiences of embedding PPI in doctoral research, from designing research to dissemination (6–10). These are useful as they help doctoral researchers understand the process of involvement and consider the resources and time required for involvement activities. However, to date, no article has described the process of involving disabled adults in the doctoral research process. Involving lived experience of disabled people in the research process is fundamental to getting insights on key issues and identifying where research needs to be directed (11, 12). However, the literature indicates that there are specific challenges with meeting access needs when involving adults with intellectual impairments in research (13, 14). Researchers must consider the person's impairment and environmental needs, and ensure there is adequate time and resources to support their involvement (13, 14). In addition, studies involving physically disabled children and young people reported that researchers need to consider limitations from physical impairments, participation needs, and the accessibility of the environment (15, 16). These issues may be challenging for doctoral researchers to address and may deter them from attempting to engage disabled adults in the research process.

Restrictions and social distancing measures, implemented as a result of the COVID-19 pandemic, have caused additional challenges in conducting PPI in research (17–19). Researchers have had to change and adapt their approaches and embrace digital technologies that allow PPI to take place remotely (17). Pivoting toward digital technologies may be both a challenge and an opportunity for researchers when involving disabled adults in research.

Cerebral palsy (CP) is a childhood-onset physical condition described as “a group of permanent disorders of the development of movement and posture, causing activity limitations.” (20) In addition to physical impairments, people with CP often experience sensory, cognitive or speech impairments. (20) Adults with CP also present with an increased risk of non-communicable diseases and mental health conditions compared to the general population (21, 22). They require ongoing coordinated health services to meet their needs (23). However, there are lack of research articles describing how their voices are included when conducting health services research.

This paper aims to provide an overview of how adults with CP were involved in a doctoral research process during the COVID-19 pandemic and the impact of their involvement on the research process. The content of this paper is informed by the GRIPP 2 checklist, which is international guidance in reporting PPI in research (24). The objectives of this paper are to:

- Describe the involvement and impact of adults with CP in each stage of a doctoral research process.
- Describe the experiences of PPI from the perspectives of adults with CP and the doctoral researcher.
- Discuss the benefits and challenges to PPI during the pandemic.

## Methods

The insights presented in this paper were obtained from the experience of conducting a doctoral research project that explored health service use among adults with CP using a mixed-methods approach. The doctoral research project included a mixed-methods systematic review (23), two quantitative studies (25) and a qualitative study. PPI was included from the outset in all elements of the project.

### Researcher's Role and Experience

The supervisory team (JR, AW, CK) has experience in PPI, particularly in the field of disability (e.g., in the Northern Ireland CP Register a new public involvement group was set up) (26). A Research Fellow (JF) who is experienced in conducting PPI meetings in the UK and Ireland was also part of the research team. This Research Fellow helped the doctoral researcher to prepare for meetings and to facilitate the meetings. The doctoral researcher (MM) is a physiotherapist by background and has clinical experience working with disabled people both in the UK and India. She had no experience of PPI prior to starting her PhD.

### Recruitment

We used snowball sampling to recruit PPI contributors from November 2019 to January 2020. PPI contributors were recruited from organizations and services known to the research team. The PPI recruitment information leaflet included a summary of the doctoral project, role of PPI contributors, person specification, remuneration, training and support, benefits of PPI, confidentiality statement, conflicts of interest and research team contact information (Supplementary Appendix 1). No geographic limitations were set within Ireland (i.e., county and area of living). The contributors were from diverse demographics (i.e., sex, age, education level and work experience) and CP characteristics (i.e., anatomic distribution of CP, and mobility level) as described in Supplementary Appendix 2. Five adults with CP living in Ireland, of both ambulatory and non-ambulatory CP, contacted us to join the PPI group and are included as co-authors (EW, FW, JG, KF, and SH).

### Data Source

Data sources used to collate the perspectives reported in this paper were: (1) a reflective diary kept by the doctoral researcher during the research process. Reflections were written by the doctoral researcher within 24 h of the meeting, or after any conversations with the PPI contributor *via* telephone, email or video call. Reflections were structured around the six questions (Table 1) that were informed by a PPI workshop attended by the doctoral researcher. (2) Four hundred fifty-four email exchanges between PPI contributors and the doctoral researcher from January 2020 to February 2022. The reasons for email exchanges between the PPI contributor and the doctor researcher were to recruit PPI contributors, arrange meetings, discuss accessibility needs, share PPI training conferences, send meeting minutes, follow up meeting action points, receive feedback from contributors about the meeting or study findings, send vouchers to contributors, and finally to update contributors about study progression, (3) meeting minutes and/or notes taken during PPI meetings. All digital meetings were recorded with consent from the contributors. The doctoral researchers used these recordings to write reflection and to help write detailed minutes. The minutes were then sent to the contributors and researcher (JF/JR) for review and was approved by the team. The recordings were deleted by the doctoral researcher within 24 h of the meeting, and (4) written reflections undertaken specifically for this paper by both the doctoral researcher and the PPI contributors. The PPI contributor's written reflections for this paper were collected between 29th of November 2021 to 20th of December 2021. The PPI contributors answered questions, as described in Table 1 *via* 31 email exchanges, and one video call arranged by the doctoral researcher.

**Table 1:** Methods of data collection and data management.

**S. No**.	**Data Source**	**Data collection**	**Data management**
1.	A reflective diary kept by the doctoral researcher during the research process	The reflection was written by the doctoral researcher within 24 h of the meeting, or after any conversations with the PPI contributor *via* telephone, email or video call. The reflection was structured around six questions: 1. What is my reflection of involvement? 2. What is my experience of conducting PPI? 3. What is my perception of PPI member's feedback or comments? 4. Who influenced different decisions? 5. What I learned from these conversations? 6. What changed as a result?
	The reflective diary was written by doctoral research since recruitment. It includes reflection on PPI recruitment process, PPI meeting experience, impact/decisions made based on PPI contributions input, reflection on discussion with supervisory and research team on PPI input throughout the research process. This reflection was written in a spreadsheet to enable searching of questions across meetings which facilitated analysis of reflections, Each column contained one of the six questions. Each row contained reflections from meeting 1–5.
2.	Email exchanges between PPI contributors and the doctoral researcher	There were 454 email exchanges between doctoral researcher and PPI contributors. The email exchange began from recruitment throughout the research process from January 2020 to February 2022. The main reason for email exchanges between the PPI contributor and the doctor researcher were to (1) arrange meetings, which includes sending polls to determine suitable meeting dates and sending meeting agendas and relevant information, (2) determine access needs to attend meetings both in person or remotely e.g., travel arrangements, wheelchair access, or troubleshooting technical issues for digital meetings, (3) share PPI training conferences for contributors to attend, (4) follow-up after meetings with minutes, or discuss meeting action points, or receive feedback from contributors about the meeting or study findings, (5) send vouchers to contributors to thank their time, and (6) update contributors about study progression	Throughout the doctoral research process, a separate folder was created for PPI email exchanges in the doctoral researcher's outlook email. This folder includes all the conversations that took place between the doctoral researcher and the PPI members The email subjects described the content of the email (e.g., meeting feedback, meeting minutes, study 1/2/3/4 updates etc.), which allowed the doctoral researcher to search the data from emails for this paper. Data related to the email exchanges was collated into a spreadsheet that included date of email exchange, email subject, brief description of the content of the email, PPI contributor's name, number of email exchanges and email data/content. The content includes data on PPI experience, decisions made, suggestions/opinions on research process, accessibility, COVID impact, recommendations on PPI process.
3.	Meeting minutes and/or notes taken during PPI meetings	The digital meetings were recorded by the doctoral researcher through an in built recorder on Zoom/MS Teams platform and was deleted within 24 h of the meeting. The recordings were used to write the doctoral researcher's reflection and to help write detailed meeting minutes. The minutes were then sent to the contributors and researcher (JF/JR) for review and was approved by the team. The meeting minutes includes 1. Contributor's reply to the following ice breaker questions at the start of the meeting. •Why do you want to contribute in this research? •What are you most looking forward to in today's meeting? •What you like about this group? 2. Contributors input when the doctoral researcher shared updates and progress made since last meeting. 3. Contributor's feedback, comments and discussions for individual study findings
	The meeting minutes were written in a word document and was shared to the team after each meetings.
4.	Written reflections undertaken specifically for this paper by both doctoral researcher and PPI contributors	Written reflections by PPI contributors were collected between 29th of November 2021 to 20th of December 2021. The doctoral researcher asked the five questions listed below. The PPI contributors were free to choose which questions they wanted to answer. 1. Why is PPI important in doctoral research? 2. What motivated you to take part in this doctoral research as PPI contributor? 3. What was your overall experience of being involved in this doctoral research? 4. Were there any benefits and challenges of being involved in doctoral research during the pandemic? 5. What are your recommendations for doctoral researchers planning to involve adults with disability in a doctoral research process?	The written reflections were gathered across 31 email exchanges between the doctoral researcher and the PPI contributors. The doctoral researcher also arranged a video call with those who wanted to share comments directly rather than emails. The digital meeting was recorded for taking notes and deleted within 24 hours.

Detailed descriptions of each data sources and data management of all data sources are included in detail in Table 1.

### Data Analysis

Data were analyzed using deductive content analysis (27–29). MM read each data source several times to develop familiarity with the material (27–29). A categorization matrix was created with three categories based on the objectives of this paper (1) Involvement and impact, (2) experiences of PPI and (3) COVID-19 impact on PPI. Data from all four sources were reviewed for content and coded to the objective categories in the matrix. Following initial coding, six sub-categories were created within category 2 (experiences of PPI) using the values of the INVOLVE Values and Principles framework (30), which is a partnership-focused framework (31) developed based on extensive literature review and service user's input (30). This framework includes six values: (1) respect, (2) support, (3) transparency, (4) responsiveness, (5) fairness of opportunity and (6) accountability to consider for good practice in PPI throughout the research cycle. Within category 3 (COVID 19 impact on PPI) two subcategories were created (1) benefits to involvement during COVID-19 and (2) challenges to involvement during COVID-19. The frequency of coding from all four data sources were tabulated for each objective category during the analysis. Microsoft Excel was used for data management. Coding was discussed with the research team (JR/JF) and disagreements were resolved by consensus.

The results were structured for each objectives as follows: (1) Involvement of adults with CP in the doctoral research process and the impact of their contributions at each stage of the research process is presented from the doctoral researcher's perspective. (2) Reflections on the experience of PPI are presented from both the doctoral researcher and the PPI contributor's perspectives. (3) The final category on the impact of covid-19 is presented from both the doctoral researcher and PPI contributor perspectives.

The recommendations proposed in this paper were developed based on the reflections from the doctoral researcher and PPI contributors, email exchanges, and digital video call discussions between the doctoral researcher and PPI contributors. The final proposed recommendations were agreed by all PPI contributors. Ethical approval was not required for this paper as the PPI contributors were active collaborators in this doctoral research rather than participants in the research (32).

## Results

### Involvement and Impact

The involvement and impact findings below were collated from (1) the doctoral researcher's reflection on the PPI recruitment process, and the impact on the doctoral research process, (2) 190 email exchanges between the researcher and the PPI contributors, on 33 different email subjects; and (3) Five meeting-minutes documents.

Public and Patient Involvement in this project was initiated by the primary supervisor but planned and coordinated by the doctoral researcher. The recruitment flier was developed by the doctoral researcher with the support of the research team (JR/JF). The PPI contributors and researchers had not engaged with each other to support the design and delivery of research previously. Funding to support PPI was included in the PhD funding that was obtained by the primary supervisor. The PhD grant-supported remuneration, travel expenses and training for the doctoral researcher. Our contributors were reimbursed for their time to attend meetings (€20 per hour) and travel costs by vouchers.

Our first PPI meeting was held face to face, at a venue that was wheelchair accessible and had good transport links thus facilitating an easy commute. The doctoral researcher arranged taxis for PPI contributors as required. At the first meeting, we discussed the terms of reference, role descriptors, preferred communication methods and preferred meeting arrangements. The terms of reference included PPI contributions at different stages of the project, ground rules on confidentiality, duration of meetings, and reimbursement. All subsequent meetings were held online because of the Covid-19 pandemic. The meetings were arranged a month in advance *via* doodle poll. A meeting agenda, presentation slides and relevant information were shared *via* email a week before the meeting. When contributors were unable to attend PPI group meetings, the doctoral researcher arranged a one-to-one phone/video call with contributors.

Public and Patient Involvement contributions and resultant impacts at the various stages of the doctoral process are described in detail in Table 2. Overall, PPI resulted in clear, accessible research information being developed and widely disseminated. Also, our PPI contributor's interpretation of findings enabled the doctoral researcher to bring research knowledge into context. PPI contributor input to the research dissemination plan helped identify key audiences and creative ways to share findings.

**Table 2:** PPI contribution and impact on the research process.

**Stages**	**PPI contribution**	**Impact on the research process**
Developing research questions	• Identified relevant factors to examine associations with health service use. • Identified relevant factors to examine associations with physiotherapy services needed and received for adults with CP. • Defined terminology “health services” and “health professional” in plain English. • Advised to change the language used from “caregiver” to “support person/people” to describe people who are both paid and unpaid carers for adults with CP.	• Helped to develop study objectives and informed the statistical analysis plan. • Identified factors of importance resulting in research findings that were meaningful and relevant to adults with CP. • This resulted in information about the study being clear from the start. • Clarity on inclusion criteria for participant recruitment in the design phase of the study.
Data collection	• Developed plain English study documentation for adults, support persons and service providers, which includes participant information sheet, invitation leaflet, consent forms and topic guide. • Developed Easy Read version for adults with CP, which includes participant information sheet, invitation leaflet and consent forms. • Developed study website www.theeachstudy.com by reviewing the study logo, study name, study video, website layout, image, theme, tabs and contents of the webpage. • Inputted on color contrast of the background to font, font size, using audio to text, links to easy read information leaflet on the home page and including PPI tab on the website. • Advised on interview adaptations for mild to moderate intellectually disabled people, and/or communication or visual impairments to take part. This included conducting joint interviews, using images in the topic guide, and easy read study documentation. • Shared study information with their contacts (adults with CP, support people and service providers); social media; and advocacy or disability groups in Ireland.	• Developing plain English or Easy Read study documentation resulted in information about the study being clear and appealing to potential participants. It also supported the consent process by ensuring that the information was understandable. • This accessible website helped us to recruit people across the country and including the contributors' video/comments on the website made it appealing for participants to take part. • Adaptations made this study accessible to people with CP who had additional impairments. • This wide dissemination helped us to recruit 43 participants including adults with CP, support people and service providers in this study.
Interpretation	• Interpreted the findings from each component study in the doctoral research project by discussing the following questions: - What do they think about the findings? - Do they agree/disagree with the findings? - What could be the possible reasons behind those findings? - What are the gaps identified in the findings? - What are the limitations of these findings?	• Helped with the discussion section of the component studies
Dissemination	• Developed dissemination plan for each component study.	• Helped identify key target audiences and organizations to share findings with so that the work reaches the target audiences and translates to change in policy and practice. • Identified research outputs to produce that would reach adults with CP, researchers, and service providers.

### Experience of PPI

The experience of PPI from the perspective of the doctoral researcher and the PPI contributor is detailed in the reflection below, where it is structured using the INVOLVE values and principles framework. (30) The experience of PPI were collated from doctoral researcher reflection notes; 42 email exchanges between the researcher and the PPI contributors; five meeting-minutes documents and reflections shared by PPI contributors for this paper.

#### Respect

The doctoral researcher respected the PPI contributor's perspectives, insights and expertise throughout the research process, where the opinions of all the contributors were considered in progressing the doctoral research.

“*From my experience, respecting and acknowledging people's time, experience, input, strengths and limitations are important in involving disabled people throughout the doctoral process.”- Doctoral researcher (MM)*.

“*My experience of being involved in this research was overall very positive. I felt happy to give my time as I knew that it was appreciated and valued”-* ***EW***

The PPI contributors also respected the lived experience of other contributors and learned from each other as a group (e.g. age-related changes in CP).

“*I have really enjoyed being part of this panel. And I've learned a lot from the other participants. So not only does PPI raise public awareness, but it benefits you personally as well because you end up sharing your story with other people in a similar situation. And sharing advice and tips on how to deal with various aspects of having cerebral palsy”-* ***SH***

In respect of their contribution to the doctoral research, PPI contributors were acknowledged in all published papers and presentations. They are also co-authors in this paper.

#### Support

The doctoral researcher was well-supported by regular training and conferences to upskill the PPI process in research, which had a positive impact on the doctoral researcher's experience with PPI.

“*My experience of PPI in this doctoral process was positive with adequate support and training from the structured PhD programme and the research team. The support and training I received included workshops on PPI and peer learning events on PPI provided by my structured PhD programme. The peer learning events allowed me to interact with other doctoral researchers who had used PPI and the challenges they faced. I also attended conferences on PPI provided by the National University of Ireland Galway, University of Limerick, and PPI Ignite seminar series-3 in Dublin City University. These learning opportunities and supports helped me to understand how PPI was applied in various research projects and to learn how to involve contributors at various stages of the research process.-Doctoral researcher (MM)”*

The doctoral researcher also shared “PPI in research” conferences with the contributors to support their training. The doctoral programme provided funding support that helped to reimburse through vouchers, arrange taxi, transport or refreshments for meetings. The PPI contributors equally appreciated the support in valuing their time and commitment.

“*A big thank you also for sending the vouchers. I very much appreciate it.”- JG*

In addition, a research partner organization supported the recruitment of PPI contributors and made accessible meeting rooms available before the pandemic.

#### Transparency

Both PPI contributors and the doctoral researcher agreed on the terms of references, role and level of involvement in research, and PhD timelines at the start. The doctoral researcher also discussed and agreed on the availability, accessibility needs, preferred method of communication, and reimbursement for time and travel arrangements in the initial meetings. This further helped to work in collaboration throughout the doctoral process and manage expectations.

“*Also, I believe being honest and open about the project aim, timelines and scope from the start has helped me and contributors to stay on track with the project timeline”- Doctoral researcher (MM)*

The PPI contributors were positive about the transparent approach in conducting this doctoral research.

“*The whole PhD project is a concrete evidence with reproducible data. The doctoral researcher is approaching this in the right way. It's great to see some evidence. There is nothing done in Ireland like this before”-* ***EW***

The doctoral researcher was transparent when arranging meetings, which was sent *via* doodle poll a month in advance to accommodate the availability of all contributors. Similarly, meeting minutes were shared with the team and agreed decisions were shared to ensure transparency.

#### Responsiveness

The doctoral researcher actively responded to the PPI contributor's feedback throughout the doctoral process. This was reflected and discussed between the doctoral researcher and the supervisor (JR).

“*Throughout the project, I took our PPI contributors' feedback seriously, which was important for building a working relationship with the contributors. For example, one of our contributor's suggested arranging individual meetings before the main PPI meeting, to provide an overview of issues that will be discussed in the group meeting. I arranged a separate meeting with one of our contributors before every group meeting, and this helped the contributor to prepare and process the information and actively involve and share views in group discussions”- Doctoral researcher (MM)*.

“*I find that overall the doctoral researcher on this project are really engaging/listening with us. It is a pleasure to be part of it”-* ***FW***

#### Fairness of Opportunity

The doctoral researcher ensured that equal opportunities were provided to all PPI contributors starting from the recruitment of contributors from different demographic groups as described in Supplementary Appendix 2. During all the meetings, the PPI contributors were given equal opportunities to speak and contribute.

“*I reflected before and after every PPI meeting and discussed the meeting agenda and meeting reflection with my supervisor (JR), which helped me to develop areas that I needed to improve in involving PPI contributors. For example, we discussed giving equal opportunity for contributors to speak in meetings, and options to contact the doctoral researcher directly following meetings. This has enabled me to ensure that all our contributors' voices were equally heard throughout the doctoral process.”- Doctoral researcher (MM)*.

“* involved because of lack of research out there. Very inclusive research and delighted to be part. Hoping that the outcomes will influence change.” – FW*

“*I like sharing ideas. It is always nice to talk to everybody in the group. The PPI meetings and talking to the doctoral researcher one to one has helped in building the confidence to interview/recruit my personal assistant”-* ***KF***

The doctoral researcher ensured that the venue selected for in-person meetings were accessible for PPI contributors. Similarly, the online platforms that were accessible to all contributors were used.

#### Accountability

The researchers and research organizations had policies in place for the governance of public involvement in research and public accountability through terms of reference. The doctoral researcher informed PPI contributors regularly of the research outcomes and the PPI impact on research, which was part of the doctoral researcher's accountability to research. The researcher learned and reflected on the PPI process by writing this paper, which the contributors reviewed and involved in writing this paper.

“*I think being able to write this paper with PPI contributors was a great learning experience on PPI process.”- Doctoral researcher (MM)*

“*the biggest challenge is how can this research influence positive change for those with CP using health care services in the future”.-****FW***

“*I wanted to take part in the PPI panel because I feel it is important for those with lived experience of healthcare services for adults with cerebral palsy in Ireland to talk about their personal experiences of accessing health services for adults with CP in order to evaluate existing services and highlight gaps in service provision while also providing suggestions for improvements to services”-* ***JG***

The doctoral researcher and PPI contributors felt accountable to share these research findings to make a meaningful impact for adults with CP accessing services.

### Benefits and Challenges to Involvement During COVID-19

The benefits and challenges to involvement in the doctoral research process during COVID-19 is described from both the doctoral researcher and PPI contributors' perspective. The data was extracted from 54 email exchanges between the doctoral researcher and the PPI contributors since the pandemic, five meeting-minute documents and reflections shared by contributors for this paper.

Public and Patient Involvement contributors and the doctoral researcher recognized that there were both benefits and limitations of conducting online PPI meetings during the pandemic. The benefits of remote meetings meant that PPI contributors could join the meetings in a comfortable environment at their home or workplace. Remote meetings also allowed some PPI contributors to attend meetings without needing to schedule their personal assistants to support commuting for in-person meetings. One PPI contributor required a support person for technical assistance. Also, in remote meetings, PPI contributors were not concerned about environmental barriers and physical accessibility challenges (e.g. wheelchair access) as they may have been if the meetings were in person. However, the transition to digital meetings was initially challenging for the doctoral researcher due to lack of experience in using digital platforms. Members of the research team (JR, JF) supported the doctoral researcher in testing various platforms (MS Teams, Zoom, Skype) and gaining confidence in hosting digital meetings.

There were some technical challenges (e.g. audio/video issues) in the initial digital meeting on the Microsoft Teams platform, so we moved to the Zoom platform for the remaining meetings. Both the PPI contributors and doctoral researcher worked closely to solve the technical problems, where the doctoral researcher arranged a test call with PPI contributors before the meeting to help resolve the technical issues. There was a benefit in resolving the technical problems with the contributors, where it increased the bonding relationship between the doctoral researcher and the PPI contributors. In addition, we felt that the physical distance in meetings limited our role in probing and facilitating discussion, or conducting group activities when compared to face to face meetings. There were also fewer opportunities to identify body language in digital meetings. However, to overcome this the second researcher acted as a research scribe (JF) and the supervisor facilitated the discussion along with the doctoral researcher. We also used debriefings, and field notes to capture all online interactions. We also used different ways to ensure that meeting information was accessible to PPI contributors remotely, which includes sharing meeting agendas, accessible slides (e.g., font size, color contrast, background theme for visual impairments) and meeting summary with clear action points for PPI contributors.

The doctoral researcher regularly updated the contributors *via* email or phone about the research project, shared progress/output from the project, and shared information on PPI training conferences in Ireland.

## Discussion

This paper highlights how adults with CP can be involved in different stages of the doctoral research process during the pandemic, with reflections from both doctoral researcher and PPI contributors. It also provides recommendations to researchers planning to involve disabled adults in the doctoral research process.

### Involvement and Impact

Public and Patient Involvement involvement in this doctoral research resulted in development of study objectives relevant to adults with CP. It is known that when research aims align with PPI contributors issues, there is increased motivation among PPI contributors throughout the research process (6–8). Furthermore, PPI contribution has consistently been shown to improve study recruitment (2, 6–9, 33). Our contributors identified effective ways to facilitate recruitment similar to those reported previously in the literature (2, 7). Although previous studies of PPI in the doctoral process involved PPI contributors in data analysis (6–9) in this doctoral research we shared the analysis plan and findings which allowed PPI contributors to review and provide feedback accordingly. Involving disabled adults in the interpretation of findings contextualized our findings which is recognized as important for knowledge translation into practice (34). Challenges in quantitatively evaluating PPI in research have been acknowledged (35–37) and studies of PPI in doctoral research note challenges in conducting formal evaluation of PPI impact due to lack of guidelines or tools (6–8). Therefore we took the approach to personally reflect on the impact of PPI contribution to a doctoral research project.

### Experience of PPI

It is well recognized that PPI requires adequate training, time, funding and resources (2, 5–8, 34). The doctoral research budget included funding for PPI ensuring that the doctoral researcher's experience was positive due to support from the supervisory team, PPI training, dedicated funding, and resources available to support reimbursements. Involving disabled adults was helpful as it enabled the doctoral researcher to get deeper insights from people with lived experience. This required consideration of disabled adults' impairment, participation, and environmental needs, as described in the International Classification of Functioning, Disability and Health (ICF) (38). However, PPI by disabled adults can be challenging for doctoral researchers working on a limited timeframe, similar to that reported in the wider literature (6–9). The doctoral researchers also need careful consideration of managing expectations of PPI contributors along with funders, supervisory team and the programme timelines. This was managed by an open and honest discussion about the timelines from the outset, which in turn helped collaborative working toward those timelines. Similar to previous reports (6–10), the experience from our PPI contributors was positive: they felt valued, listened to, empowered and motivated to be involved.

### Benefits and Challenges of Involvement During COVID

The remote meetings during the pandemic were flexible for our PPI contributors in terms of time, environment and location (17) as all had access to the necessary technology, however, this may not be the case for all potential PPI contributors (39). Following initial minor technical issues, remote meetings provided equal opportunity for all our contributors to be involved. It has been reported that online platforms can provide anonymity for contributors to express their views (8), however, our PPI contributors were comfortable sharing their views regardless of remote or in-person meetings.

### Recommendations

Our proposed recommendations are described in Table 3. However, future research is needed to develop recommendations using a rigorous process that incorporates the views of all key stakeholders. The recommendations proposed may provide practical suggestions on involving disabled adults in doctoral research. The doctoral researcher learned ways to make the study process accessible from PPI contributors. When involving disabled people, it is recommended to share accessible findings with contributors (13). In addition to sharing accessible findings our paper describes the importance of making meetings accessible both in-person and remotely. Although, there is a lack of guidance on how representative PPI need to be in research (40), we recruited contributors from diverse age range, sex, county of living, and types or severity of CP. We recommend involving diverse and marginalized voices throughout research process. We also recommend doctoral researchers, and supervisory teams, looking to involve disabled adults in doctoral research to recognize the common challenges described in this paper (e.g., Funding, accessibility, resources, time, and training needs) in the planning phase of the doctoral project.

**Table 3:** Recommendations when conducting PPI in doctoral research with adults with CP.

	**Recommendations**
1. Recruit people with CP through networks or organizations that support the population studied	- Be open in to involving marginalized voices in research by recruiting people with CP. - Share recruitment information to organizations and networks that support people with CP to help recruitment. Use the snowball sampling method if required. - Set timeline for PPI recruitment in the planning phase, this helps to stay focused on PhD timeline . - Respond to any queries related to taking part from adults with CP or similar disability immediately.
2. Plan PPI when designing or developing research questions in doctoral research	- Identify research question that is relevant for people with CP . - Involve people with lived experience of disability in doctoral research when developing a research question. - Secure additional funding to conduct PPI in doctoral research and if needed apply additional small grants for PPI. - Regular training for both researchers and adults with CP on PPI should be embedded in the doctoral research process. - Allow adequate time for PPI in doctoral research by embedding it within the PhD timeline. - Involve PPI contributors to develop study documentations in plain English and Easy read versions to increase accessibility. - Pilot topic guides with PPI panel contributors and refine interview questions accordingly.
3. Plan PPI meetings well in advance and make necessary adaptations to involve people with CP	- Consider accessibility needs of adults with CP and make necessary adaptations as required to attend meetings. Also, provide options to attend the meeting with a support person if needed. - Make travel arrangements that are wheelchair accessible for adults with CP to attend meetings in person. - Arrange a one-to-one meeting with the PPI contributors if required to summarize what will be discussed in the group meeting beforehand. - Send doodle poll and meeting agenda/information before the meeting, so the contributors are fully informed during meetings. - When using an online portal for meetings, identify a portal (e.g., Zoom) that suits all contributors, and arrange test calls if needed before the meeting. - Discuss terms of reference, role descriptors, preferred communication methods, and reimbursement in the initial meeting to meet any expectations and refer back to them when needed. - Before all meetings discuss meeting contents with the research team, e.g., review terminology/medical jargon, and structure of meetings. - One researcher should take notes during the meeting and document every input from PPI contributors at various stages of research. Maintain a reflective journal for all PPI meetings.
4. Involve PPI contributors throughout the research process and regularly update PPI contributors on the research progress	- Be open to taking PPI contributors input throughout the research process and acknowledge their expertise. - Discuss all findings with panel contributors and document their interpretation. - Follow values and principles framework throughout the doctoral process and document how it was incorporated. - Build a trusted relationship with adults with CP throughout the research process. - Be transparent to the PPI contributors about the research progress regularly. - Regularly update PPI contributors on research training opportunities available.
5. Discuss dissemination plan throughout the doctoral research process	- Record impact of PPI on research design, research ethics, PPI contributors, researchers, research participants, wider community or organization and implementation throughout the doctoral research process. - Discuss dissemination plan for individual findings and identify target audiences with the PPI contributors. - Be open to PPI contributors creative ideas of sharing findings (plain English/Easy read leaflets, infographics, podcast, video, research brief, presentations, and journal publications) throughout the doctoral research cycle. - Involve PPI contributors to co-present the findings and co-write the papers.

## Conclusion

Public and Patient Involvement by adults with CP in this doctoral research was reported to have a positive impact on the research process, and the experiences were positive, despite COVID challenges. The recommendations from the doctoral researcher and adults with CP may guide future researchers who are planning to involve disabled adults in their work.

## Data Availability Statement

The original contributions presented in the study are included in the article, further inquiries can be directed to the corresponding author.

## Ethics Statement

Ethical review and approval was not required for the study on human participants in accordance with the local legislation and institutional requirements.

## Author Contributions

MM, JR, and JF contributed to conception and design of this paper. MM collated the data source and wrote the first draft of the manuscript. MM, KF, JG, SH, EW, and FW wrote the reflection in this paper. JR, JF, CK, and AW provided feedback on the first draft of the manuscript. All authors contributed to manuscript revision, read, and approved the submitted version.

## Funding

This work was conducted as part of the SPHeRE Programme under Grant No. SPHeRE/2018/1. This study is funded by the Royal College of Surgeons in Ireland (RCSI) through the StAR programme.

## Conflict of Interest

The authors declare that the research was conducted in the absence of any commercial or financial relationships that could be construed as a potential conflict of interest.

## Publisher's Note

All claims expressed in this article are solely those of the authors and do not necessarily represent those of their affiliated organizations, or those of the publisher, the editors and the reviewers. Any product that may be evaluated in this article, or claim that may be made by its manufacturer, is not guaranteed or endorsed by the publisher.
