# Supplementary material for: Public and Patient Involvement in Doctoral Research During the COVID-19 Pandemic: Reflections on the Process, Challenges, Impact and Experiences From the Perspectives of Adults With Cerebral Palsy and the Doctoral Researcher
Source: Front Rehabil Sci. 2022 Jun 3;3:874012. doi: 10.3389/fresc.2022.874012 (PMC9397843; doi:10.3389/fresc.2022.874012)
Supplement: Supplementary file 1 [file Data_Sheet_1.PDF]

Exploring health service use and the related experience among adults with cerebral palsy

Public and Patient Involvement Panel

We are establishing a Public and Patient Involvement (PPI) panel to advise on a research project exploring health service use and the related experience among adults with cerebral palsy (CP) in Ireland. The role of the PPI panel is to work with the research team to plan, design and manage this project, and to evaluate and share the research findings.

We are seeking adults with CP (≥ 18 years) in Ireland to sit on the PPI panel.

Summary of project

The aim of this research is to explore health service use among adults with CP and the related experiences. To achieve this, we will firstly summarise findings from previous research that has examined the proportion of adults with CP using health services, the frequency of health service use, and the experiences of health service use from the perspective of adults with CP, caregivers and health professionals. Secondly, we will identify the patterns of health services used and the unmet needs of adults with CP in Ireland from the National Physical and Sensory Disability Database (NPSDD) data. The NPSDD is a voluntary database of people with disabilities that includes data on therapeutic, specialist, assistive or support services and unmet needs, up to 65 years in Ireland. Thirdly, we will conduct a survey examining experiences of physiotherapy services among adults with CP in the UK and Ireland. This survey will look at how they access services, their reasons for seeing a physiotherapist, the type of physiotherapy services they use, what they value and what could be improved. Finally, we will conduct interviews exploring the experiences of health services in Ireland from the perspectives of adults with CP, support person and health professionals.

Role of Study Steering Group Member

The overall responsibility of a member is to attend meetings and work with the research team to plan, design, manage, evaluate and share the research.

Specific duties and responsibilities may include the following:

- To attend approximately 10 meetings over 3 years
- To support the development of documents for the project such as advertisements and information sheets
- To advise on the methods used to recruit participants
- To advise on the methods used to collect data
- To pilot methods of data collection
- To discuss and comment on the findings of the research
- To advise on, design and develop leaflets and other materials outlining the findings of the research for adults with CP, caregivers and health professionals

Time commitment

The PPI panel will meet approximately three times in 2020, 2021 and 2022. Meetings will last approximately 2 hours.

Meeting attendance:

Meetings will be held at RCSI, the Central Remedial Clinic or a location convenient to all members. Members will select the most suitable time to hold the meetings, which may include evenings or weekends if requested.

Person specification:

Members should:

- Be willing to engage in discussions with the group and share ideas
- Be willing to provide critical feedback
- Have access to and be willing to communicate with the research team by email or telephone to arrange meetings
- Be able to attend meetings. Support will be provided to attend meetings if required. Although the preference is for members to attend meetings in person, members will be able to attend via phone if they cannot attend in person.
- Have an interest in learning more about the research process

No previous experience or knowledge relating to research is required to be a member.

Remuneration

Members will receive €20 per hour for their time to attend the meetings in a voucher of their choice. Members will also be reimbursed for their travel expenses. Refreshments will be provided.

Training and Support

The project leads will endeavour to:

- Support members of the group to identify any training needs
- Provide relevant information and answer outstanding questions
- Support members to contribute before or after meetings if they are unable to participate during meetings

Benefit

Benefits of membership of the group may include:

- Ensuring your perspectives on health services for adults with CP services are included in research.
- Sharing your experience and learning from other people's experiences
- Ensuring research findings are shared with adults with CP, families and health professionals

Confidentiality

Some discussions or papers circulated for meetings may be considered confidential. If unclear, please seek clarification from the research team. By becoming a member of the PPI panel you agree to uphold this confidentiality

Conflicts of interest

As with all members of a research team, members of the PPI panel will be required to disclose any involvement with individuals, government bodies, commercial interests or third parties, which could lead to a conflict of interest with the work of the group. A conflict of interest does not stop someone from becoming a PPI panel member but must be declared.

Research Team

Project Lead: Manjula Manikandan, RCSI.
Research team: Jennifer Ryan, RCSI; Jennifer Fortune, RCSI; Aisling Walsh, RCSI; Claire Kerr, Queens University Belfast.

Project Funding: Funding for this project is provided by the Royal College of Surgeons in Ireland.
