# Supplementary material for: Public and Patient Involvement in Doctoral Research During the COVID-19 Pandemic: Reflections on the Process, Challenges, Impact and Experiences From the Perspectives of Adults With Cerebral Palsy and the Doctoral Researcher
Source: Front Rehabil Sci. 2022 Jun 3;3:874012. doi: 10.3389/fresc.2022.874012 (PMC9397843; doi:10.3389/fresc.2022.874012)
Supplement: Supplementary file 2 [file Data_Sheet_2.PDF]

Appendix 2. PPI contributors demographics (n=5)

		n
**Age**	18-30 years	2
	31-40 years	1
	41-50 years	
	51-60 years	2
**Sex**	Male	1
	Female	4
**County**	Dublin	1
	Cork	2
	Meath	1
	Waterford	1
**Living area**	Urban	5
	Rural	0
**GMFCS level***	I	
	II	
	III	4
	IV	1
	V	
**Employment status**	Full-time	1
	Part-time	3
	Not working/ Unemployed	1
	Retired	
**Education level**	Primary level education	1
	Second-level education	
	Undergraduate level education	1
	Postgraduate level education	3

*GMFCS: Gross Motor Functional Classification System, where level I-II are ambulatory and III-V are non-ambulatory
